# Simultaneous Presentation of Relapsed Diffuse Large B-cell Lymphoma and Extrapulmonary Tuberculosis in a Patient With HIV: A Case Report

**DOI:** 10.7759/cureus.78263

**Published:** 2025-01-30

**Authors:** Toshali Pandey, Sumant Inamdar, Susanne Jeffus, Soumya Pandey, Ankur Varma

**Affiliations:** 1 Internal Medicine, University of Arkansas for Medical Sciences, Little Rock, USA; 2 Gastroenterology, University of Arkansas for Medical Sciences, Little Rock, USA; 3 Pathology, University of Arkansas for Medical Sciences, Little Rock, USA; 4 Hematology and Medical Oncology, University of Arkansas for Medical Sciences, Little Rock, USA

**Keywords:** anti-hiv agents, diffuse large b cell lymphoma, extrapulmonary tuberculosis, “hiv”, "interferon-gamma release tests"

## Abstract

Human immunodeficiency virus (HIV) infection is a strong risk factor for diffuse large B-cell lymphoma (DLBL) and tuberculosis. Both DLBCL and tuberculosis can have remarkably similar clinical presentations, proving to be a diagnostic and therapeutic challenge. We report the only known case of an HIV-infected individual who presented simultaneously with relapsed DLBCL in the form of spinal cord involvement and tuberculosis of the mesenteric lymph nodes. This case highlights the possibility of multiple co-existing diagnoses in HIV, and the need for a low threshold to obtain confirmation via biopsy. The interferon-gamma release assay (IGRA) has low sensitivity in detecting tuberculosis in patients with HIV. Adherence to antiretroviral therapy (ART) is crucial in achieving and maintaining remission in DLBCL.

## Introduction

HIV infection and non-Hodgkin’s lymphomas have a strong association. Diffuse large B cell lymphoma (DLBCL) is the most common hematological malignancy seen in people with HIV [1]. In the pre-anti-retroviral therapy (ART) era, the relative risk of DLBCL was reported to be 100-200 times greater in people with HIV than in the general population [1]. Advances in ART have reduced the incidence of DLBCL in people with HIV; however, the risk continues to be elevated and may depend on the degree of CD4 T cell recovery following viral suppression [2,3]. Lymphoma can be the presenting feature of HIV and is more likely to be extranodal at presentation compared to non-HIV-infected individuals [2]. 

Tuberculosis is the leading cause of death and hospitalization among people with HIV worldwide. In people with HIV, tuberculosis progresses more rapidly and often has systemic dissemination and extrapulmonary involvement, especially with lower CD4 counts [4]. Post-COVID-19 pandemic, the United States has seen a small rise in the incidence of tuberculosis [5]. About 8000 cases were reported in 2022, of which 4.3% had HIV co-infection [5]. 

DLBCL and tuberculosis share many clinical and imaging features. Initial presentation in both conditions can take the form of constitutional or B symptoms - weight loss, fever and night sweats. Lymphadenopathy is a characteristic feature of both conditions and can virtually involve any lymph node group. Central nervous system (CNS) involvement is rare with relapsed DLBCL but portends a poor prognosis [6]. CNS involvement is also seen in tuberculosis and is five times more likely in people with HIV compared with people without HIV [7]. We report the only known case of an HIV-infected individual who presented simultaneously with relapsed DLBCL and tuberculosis, presenting a diagnostic and therapeutic challenge. 

## Case presentation

**Figure 1:**
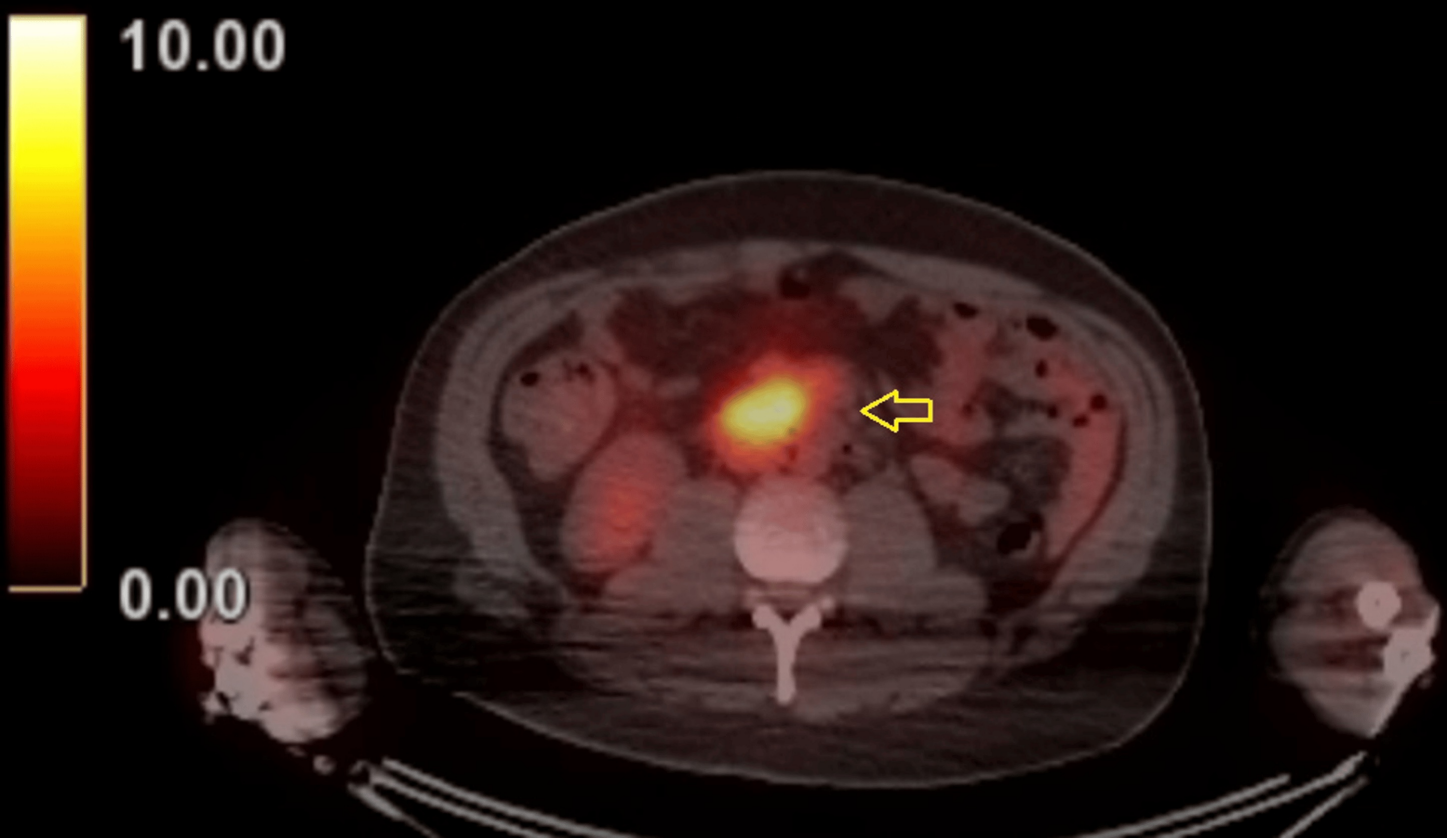
PET-CT scan demonstrating intensely FDG avid central mesenteric lymph node conglomerate (arrow) measuring approximately 2.9 x 2.3 cm in maximal transaxial dimension, SUV 5.6. PET-CT, Positron emission tomography combined with computed tomography; FDG, fluorodeoxyglucose; SUV, standardized uptake value.

A 34-year-old male patient with HIV and a history of stage IV DLBCL was admitted with acute onset progressive lower extremity weakness and back pain. He had been diagnosed with HIV about 1.5 years prior to admission and had been intermittently taking ART. He was diagnosed with DLBCL about six months prior to admission after he was found to have retroperitoneal lymphadenopathy. His serum interferon-gamma release assay (IGRA) was negative. He underwent six cycles of chemotherapy with dose-adjusted rituximab, etoposide, prednisone, vincristine, cyclophosphamide, and doxorubicin (R-EPOCH) and four cycles of intrathecal methotrexate for CNS prophylaxis. On his last re-staging positron emission tomography (PET) scan, performed after his fifth chemotherapy cycle, he was in near-complete remission. Following chemotherapy, he was briefly lost to follow-up due to incarceration. About three weeks prior to his admission, he was seen in the clinic and found to be losing weight and suffering from fatigue. He underwent a PET-CT scan that showed a large mesenteric lymph node conglomerate (Figure 1).

Due to concerns of relapsed DLBCL, this mass was biopsied via endoscopic ultrasound-guided fine-needle aspiration around two weeks prior to admission. The results were pending.

**Figure 2:**
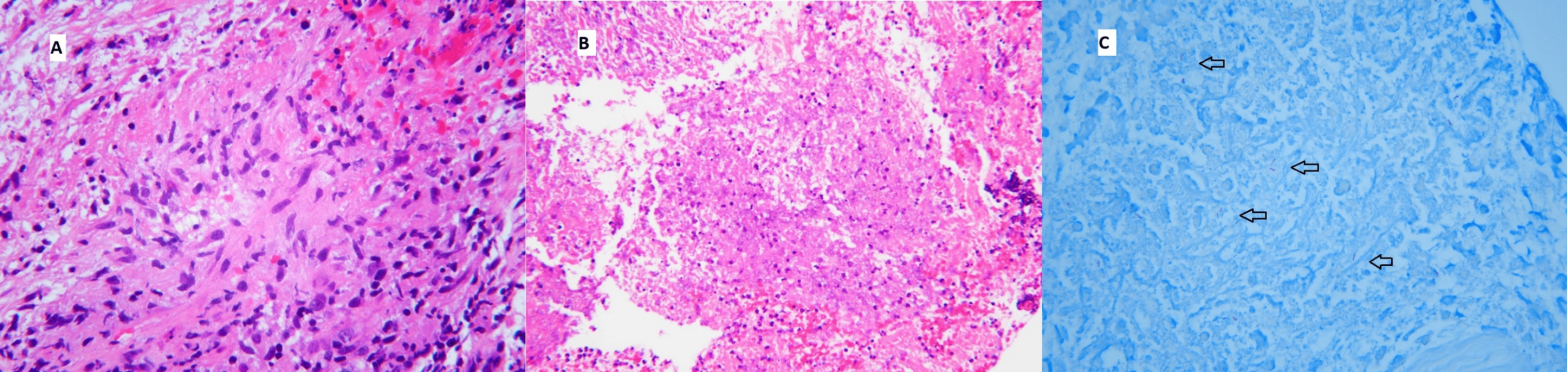
Biopsy of mesenteric lymph node mass. A. The biopsy showed areas of epithelioid histiocytes consistent with granulomatous inflammation (hematoxylin and eosin (H&E) stain, 400x magnification). B. The majority of the biopsy was composed of abundant necrosis. The necrosis contained degenerating neutrophils (H&E stain, 200x magnification). C. A Ziehl-Neelsen stain showed numerous acid-fast positive organisms (arrows), morphologically compatible with mycobacteria (acid-fast bacilli (AFB) stain, 400x magnification).

In his current presentation, he reported being fully ambulatory to turning wheelchair dependent in the span of a week. On examination, he was found to have asymmetrical bilateral lower extremity weakness, sensory loss, urinary retention and poorly localized midline lower back pain. He did not have a fever and was hemodynamically stable. His CD4 count was 114 with a viral load of more than one million copies per mL. The results of his mesenteric lymph node biopsy came back as necrotizing granulomatous inflammation with acid-fast bacilli and *Mycobacterium tuberculosis* on polymerase chain reaction (PCR) (Figure 2).

**Figure 3:**
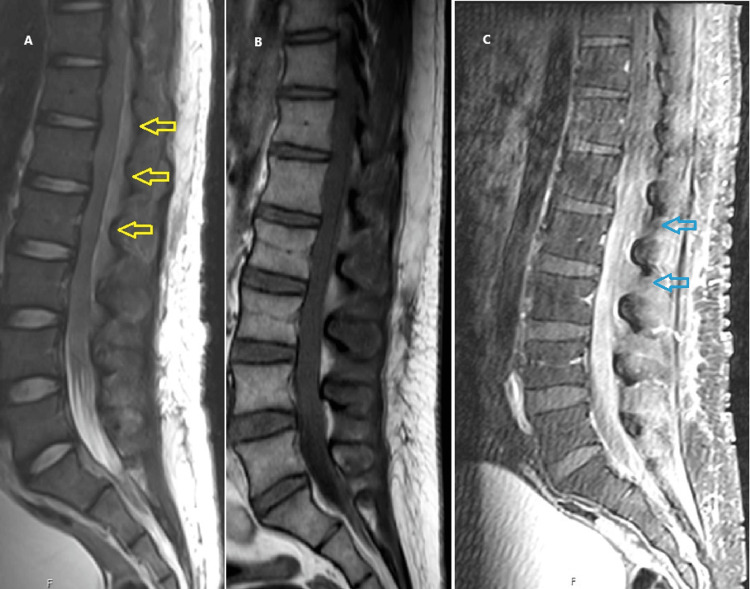
Sagittal sections from a contrast enhanced MRI of the lumbar spine showed a circumferential expansion of the epidural space from T11 to L4 (T1 hypointense and intermediate T2 signal with slight enhancement). This was noted to be exerting mass effect upon the thecal sac. There was also subtle enhancement of the nerve roots. A. Sagittal T2-weighted sequence (yellow arrows indicate epidural space expansion). B. Sagittal T1-weighted sequence. C. Sagittal post-contrast T1-weighted sequence (blue arrows indicate nerve root enhancement).

At this time, his presentation now shifted from being suspicious of relapsed DLBCL to that of CNS tuberculosis. To investigate his neurologic deficits, he had an MRI of his spine performed that showed a lumbar epidural mass along with nerve root thickening and slight contrast enhancement (Figure 3).

**Figure 4:**
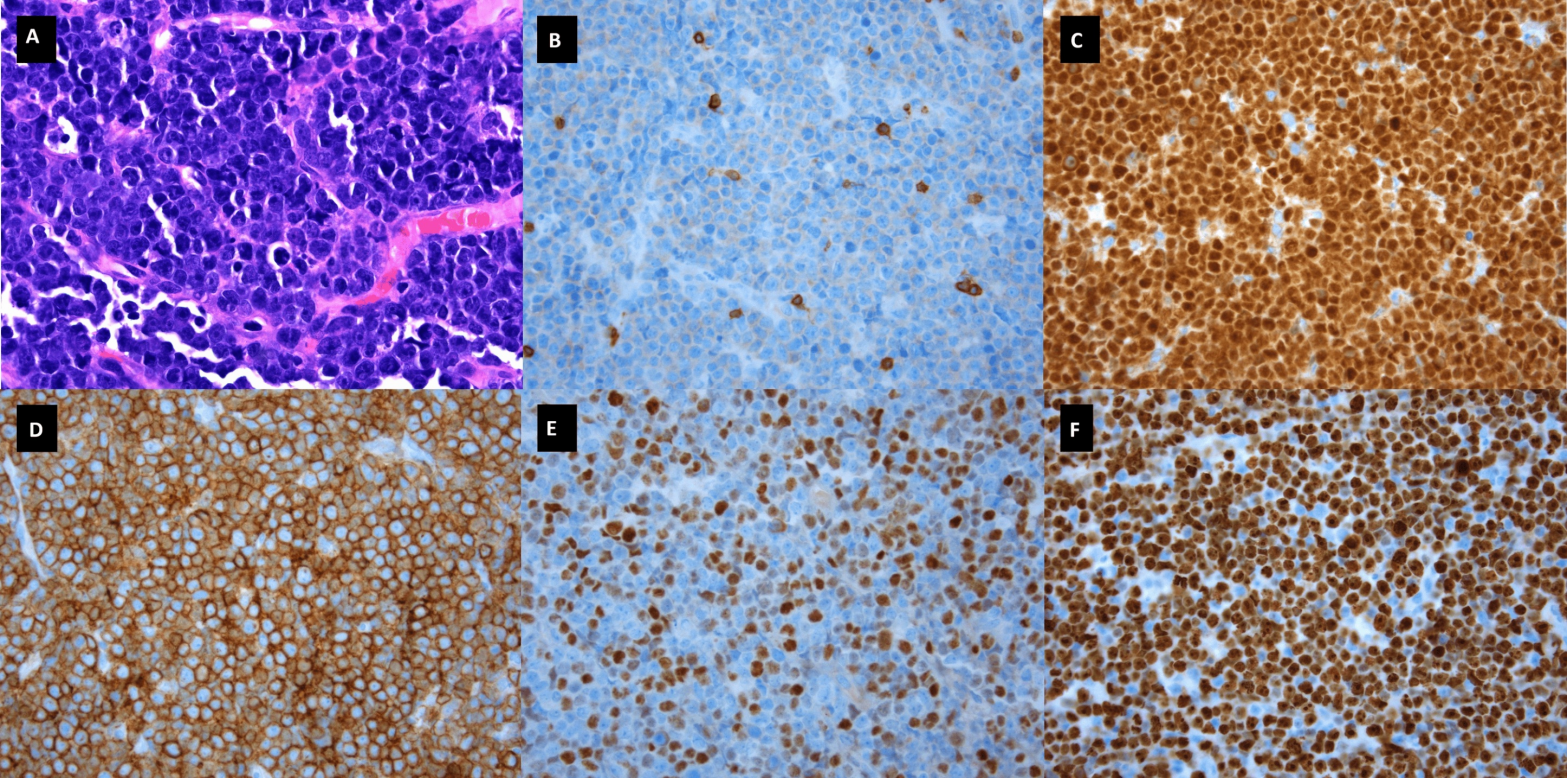
Biopsy of lumbar epidural mass. A. Histologic sections showed a neoplastic lymphoid infiltrate composed of intermediate to large-sized cells with irregular nuclear contours, and inconspicuous to small prominent nucleoli (1000x magnification). B. The neoplastic cells were negative for CD3. C. Positive for PAX5. D. Positive for CD10. E. Positive for BCL6 (subset). F. Ki-67 showed high proliferative activity >95%. B-F at 400x magnification.

He underwent emergent decompressive laminectomy. Intra-operatively, the mass was revealed to be extradural fatty tissue-looking material. The biopsy revealed sheets of cells positive for CD10, CD19, CD20, with Ki-67 positivity in >90% cells and a subset expressing BCL-6 and MYC rearrangement without the t (14;18) or 3q27 breakpoint translocation (Figure 4).

The lymphoid cells were negative for the Epstein-Barr virus-encoded RNA. This was diagnosed as aggressive diffuse large B-cell lymphoma with germinal center immunophenotype. This tumor was of identical immunophenotype and cytogenetic aberrancies as his first presentation.

Thus, he had simultaneous CNS relapse of DLBCL as well as extrapulmonary tuberculosis. He was started on anti-tubercular therapy with a combination of rifampin, isoniazid, pyrazinamide, and ethambutol. For his DLBCL, he had an Ommaya reservoir placed and was started on R-Hyper-CVAD (rituximab, cyclophosphamide, vincristine, doxorubicin, dexamethasone, intrathecal methotrexate/cytarabine alternating with high-dose methotrexate + cytarabine).

Unfortunately, he did not respond well to chemotherapy and had progressive CNS disease. His functional status declined considerably and after several ICU admissions, he opted for palliation only. He passed shortly thereafter. The patient was survived by his mother who provided informed consent for the present study.

## Discussion

This case is about a young individual with uncontrolled HIV who presented simultaneously with two conditions that can greatly mimic each other. It is the only case to our knowledge with DLBCL and active tuberculosis at the same time in an individual with HIV. Thus, we propose that the clinician should harbor a high index of suspicion for more than one diagnosis and pursue a biopsy in patients with HIV wherever possible. This includes first and subsequent presentations of what may appear to be the same disease process.

It is important to note that the IGRA performed at the time of his DLBCL diagnosis was negative. While it is possible that the patient contracted tuberculosis after the first presentation, it is likely that he may have had latent tuberculosis that was re-activated by chemotherapy and steroids. A meta-analysis by Chen et al. concluded that the sensitivity of IGRA for active TB infection in patients with HIV was only 63.1% (95% confidence interval (CI) 52.3, 72.7) [8]. They also reported a sensitivity of 64% in latent tuberculosis. However, these results come with the caveat that there was no gold standard to compare against. This is lower than the sensitivity reported for active tuberculosis in non-HIV-infected people (73-83%) [9]. Thus, the IGRA must be considered a data point rather than a definitive test to rule out latent or active TB in HIV-infected individuals.

Lastly, the prognosis for DLBCL in people with HIV is similar to those without HIV despite having more high-risk features at presentation [2,10]. This is due to the advancements in ART, resulting in fewer adverse reactions when combined with chemotherapy allowing co-administration. More importantly, immunologic recovery associated with ART has consistently improved outcomes in HIV-related lymphomas [11,12]. Interestingly, a case report has documented spontaneous regression of refractory DLBCL in a patient with HIV who had immunologic recovery on ART [13]. This implies that ART is critical to achieving and maintaining remission with DLBCL in HIV.

The generalizability of our conclusions is influenced by factors unique to our case, namely, adherence to ART, history of environmental exposure, DLBCL subtype, and lack of repeat IGRA testing at the time of confirmed active tuberculosis.

## Conclusions

We presented a rare case of concomitant DLBCL and tuberculosis in a patient with HIV that demonstrates the degree of clinical and imaging overlap between the two. A high index of suspicion and low threshold to biopsy is recommended. IGRA does not reliably rule out tuberculosis, especially in patients with HIV. ART has an important role in achieving and maintaining remission with DLBCL in HIV.

## References

[1] Kimani SM, Painschab MS, Horner MJ, Muchengeti M, Fedoriw Y, Shiels MS, Gopal S (2020). Epidemiology of haematological malignancies in people living with HIV. Lancet HIV.

[2] Huguet M, Navarro JT, Moltó J, Ribera JM, Tapia G (2023). Diffuse large B-cell lymphoma in the HIV setting. Cancers (Basel).

[3] Diamond C, Taylor TH, Aboumrad T, Anton-Culver H (2006). Changes in acquired immunodeficiency syndrome-related non-Hodgkin lymphoma in the era of highly active antiretroviral therapy: incidence, presentation, treatment, and survival. Cancer.

[4] Meintjes G, Maartens G (2024). HIV-associated tuberculosis. N Engl J Med.

[5] CDCTB: Reported TB in the U.S (2024). Reported tuberculosis in the United States, 2022. Centers for Disease Control and Prevention. https://www.cdc.gov/tb/statistics/reports/2022/national_data.htm.

[6] Ghose A, Elias HK, Guha G, Yellu M, Kundu R, Latif T (2015). Influence of rituximab on central nervous system relapse in diffuse large B-cell lymphoma and role of prophylaxis - a systematic review of prospective studies. Clin Lymphoma Myeloma Leuk.

[7] Berenguer J, Moreno S, Laguna F (1992). Tuberculous meningitis in patients infected with the human immunodeficiency virus. N Engl J Med.

[8] Chen H, Nakagawa A, Takamori M (2022). Diagnostic accuracy of the interferon-gamma release assay in acquired immunodeficiency syndrome patients with suspected tuberculosis infection: a meta-analysis. Infection.

[9] World Health Organization Use of the TST and IGRAs for the diagnosis of TB disease. WHO Consolidated Guidelines on Tuberculosis: Module 3: Diagnosis - Tests for Tuberculosis Infection.

[10] Besson C, Lancar R, Prevot S (2017). Outcomes for HIV-associated diffuse large B-cell lymphoma in the modern combined antiretroviral therapy era. AIDS.

[11] Hoffmann C, Wolf E, Fätkenheuer G (2003). Response to highly active antiretroviral therapy strongly predicts outcome in patients with AIDS-related lymphoma. AIDS.

[12] Antinori A, Cingolani A, Alba L (2001). Better response to chemotherapy and prolonged survival in AIDS-related lymphomas responding to highly active antiretroviral therapy. AIDS.

[13] Birendra KC, Afzal MZ, Wentland KA, Hashmi H, Singh S, Ivan E, Lakhani N (2015). Spontaneous regression of refractory diffuse large B-cell lymphoma with improvement in immune status with ART in a patient with HIV: a case report and literature review. Am J Case Rep.

